# Studies of an alveolar soft tissue sarcoma.

**DOI:** 10.1038/bjc.1965.86

**Published:** 1965-12

**Authors:** F. A. Udekwu, R. J. Pulvertaft

## Abstract

**Images:**


### STUDIES OF AN ALVEOLAR SOFT TISSUE SARCOMA

F. A. O. UDEKWU AND R. J. V. PULVERTAFT

*From the Departments of Surgery and of Pathology, University College Hospital, Ibadan, Nigeria*

Received for publication July 22, 1965

IN no field of oncology is there so much controversy, misunderstanding and confusion as in the field of soft tissue tumours. There are many reasons for this. First there is a great difficulty in classifying these tumours. For instance in the series of 717 soft somatic sarcomas reported by Pack and Ariel (1958) 261 or 36·4 % could not be classified into any histological type despite the fact that such excellent pathologists as James Ewing, Frank Foote, Fred Stewart, and George Higgins studied them. One might argue that these tumours are so anaplastic that de-differentiation is predominant and this would account for their poor histological characteristics. The biological behaviour of these tumours would argue against this ; for instance the overall 5-year survival of the above group was 40 %.

One of the groups of these unclassified tumours is the so-called alveolar soft-part sarcomas described first by Christopherson, Foote and Stewart in 1952. These tumours are characterized by (1) the presence of a slow growing tumour in one of the extremities, (2) the patient is usually a young adult more often a female, (3) the tumour is slow growing but definitely malignant. Therapy is still uncertain because of the lack of understanding of their histogenesis. Only by complete study of each of these rare tumours can we ever hope to gather enough information to guide their treatment and prognosis. This case is reported for both its clinical picture and the added feature of tissue culture which throws further light on the histogenesis albeit in a negative sense.

### CASE REPORT

A 26-year-old Nigerian male (UCH 132902) was admitted to the Thoracic Surgical Unit of the University College Hospital, Ibadan, on February 24, 1965, with a one-year history of a lump in the right upper arm and recurrent bouts of haemoptysis.

The tumour was at first painless but in September, 1964, it became troublesome enough to seek medical care. A doctor tried to excise it but abandoned the attempt when heavy bleeding occurred. One month later the first episode of haemoptysis occurred and although not severe lasted three days.

In January, 1965 another attempt at excision was made in the outside hospital only to be abandoned again because of excessive bleeding. Haemoptysis occurred again one month later. The lump then became painful and for the first time mild bilateral chest pain occurred. At this time he was referred to us with a suspicion of a " sarcoma " with metastases.

### *Physical examination*

The patient was superficially quite fit. The blood pressure was 150/80 mm. Hg but subsequently was repeatedly 120/80 and the raised initial systolic pressure was judged to be due to nervousness. The pulse was 90 per minute, respiration 24 per minute and temperature 37° F. There was no lymphadenopathy. The most significant finding was a lump 7 × 6 × 2 cm. on the posterolateral aspect of the right upper arm occupying most of the triceps muscle and extending from just above the olecranon to the mid arm. There was a healed scar over the central portion of the tumour without any evidence of transplanted tumour nodules. The mass itself felt rather cystic, pulsatile and warm. A bruit was heard over the lesion by several observers. In the abdomen a large non-tender liver was felt extending 7 cm. below the costal margin, but there was no irregularity. The tip of the spleen was palpable. There were no nodules on the genitalia and the prostate was normal.

*Investigations*

1. Chest X-ray showed multiple cannon ball metastases.
Skeletal survey was negative.
Intravenous pyelogram was normal.
2. Haemoglobin—14·9 g. %. PCV—43 %. MCHC—34 %. WBC—6900 with normal differential.
3. Serial sputum specimens were examined for acid fast organisms, predominant flora, fungi and malignant cells. All were negative.
4. *Liver function tests*:
Thymol turbidity—0·8 units.
Flocculation test—negative.
Total bilirubin—0·6 mg./ml.
Alkaline phosphotase—8 King Armstrong Units/100 ml.
Serum G.O.T. 14 Caband units/mol. of serum.
" G.P.T. 4 " " " "

### *Progress*

In order to make an accurate diagnosis the lesion on the arm was excised using tourniquet. It was embedded in the triceps humeri but seemed well encapsulated. It jutted against the bone but had not invaded it. It was dark, red and fleshy with clots. The histological report was of a paraganglioma and because of this diagnosis the problem of the metastatic nodules in the lungs was raised. After much consultation the lung was biopsied. Histologically the lesion in the lung was a replica of the arm lesion. A full course of nitrogen mustard (0·2 mg./kg./body-weight daily for 4 days) was given. The patient responded well and has been free of haemoptysis ever since.

### *Laboratory Investigations*

The tumour was of spherical outline, diameter 3 inches. The colour was somewhat like that of the voluntary muscle but it also had a slight yellow tint. It appeared to be well capsulated. A report from Dr. A. R. Mainwaring on the histology was as follows :—" Sections show a tumour of uniform pattern consisting of ' tubular ' structures lined by cuboidal cells, with basal nuclei and abundant eosinophilic granular cytoplasms. In places the ' tubular ' lumen is filled with such cells. Generally there is little intervening stroma, but in parts there are cleft-like endothelial lined spaces between tubules. Infiltration of muscle is not obvious. In the sections examined there is a wide band of fibrous connective tissue between muscle and tumour. Diagnosis—Paraganglioma."

The sections were forwarded for comment to Professor D. F. Cappell, University of Glasgow, who reported " This is a splendid example of that curious tumour which often goes under the eponym of Christopherson's alveolar soft-part sarcoma and which has been identified on rather inconclusive grounds I think with chemodectoma and the carotid body group. They have commonly been confused in the past with granular cell myoblastoma but they differ from it in a number of fundamental ways. My diagnosis, therefore, would be the rather less specific one of alveolar soft cell sarcoma since I am not convinced that they are true ' chemodectomata '."

A fortnight later a biopsy of lung was received and reported on by Dr. A. R. Mainwaring as follows :—" In the parenchyma there are at least a dozen rounded and discrete whitish yellow nodules, the largest 7 mm. in diameter. Sections. The histological appearances of the nodules are identical to those seen in the lesion from the triceps muscle."

Fig. 1 shows the appearance of the primary tumour. The alveolar arrangement and the vascular clefts lined by a single layer of endothelial cells are plainly shown. Fig. 2 is a higher magnification to show the granular cytoplasm, with peripherally placed nuclei. When embedded in agar and ester wax and stained by Wiggleworth's (1959) osmic acid ethyl gallate method the foamy cells are seen to contain mitochondria, but no lipoid material (Fig. 3). By this technique retraction of cytoplasm was prevented and the cells were seen to fill the "alveoli" completely.

When stained by Hortega-Penfield (Fig. 4) there was no evidence of oligodendroglia. Nuclei alone were stained but some evidence of alveolar arrangement remained.

*Tissue culture*

The tissue was minced with scissors, washed in phosphate buffer saline, and cells were dispersed at 37° C. with 0·25 % trypsin. With most soft tissue tumours

EXPLANATION OF PLATES

FIG. 1.—Primary tumour. ×400. H. & E. Formalin fixation, Paraffin embedding.
FIG. 2.—Primary tumour. ×1000. H. & E. Formalin fixation, Paraffin embedding.
FIG. 3.—Primary tumour. ×1000. Wigglesworth's method. Agar & Ester wax embedding.
FIG. 4.—Primary tumour. ×400. Hortega's method.
FIG. 5.—Haemangioma of nasal cavity. Tissue culture. Fibroblasts and tissue mast cells. ×1000. Phase. Note. Mast cells were absent in tumour of triceps ; they are said to be numerous in chemodectoma.
FIG. 6.—Haemangioma of nasal cavity. Tissue culture. Fibroblasts. ×1000. Stain—Wigglesworth's method.
FIGS. 7, 8, 9.—Tissue culture. Cells with large spherical granules. ×1000. Phase.
FIG. 10.—Alveolar sarcoma. Tissue culture. Large cell from early growth. ×1000 stain. Wigglesworth's method.
FIG. 11.—Alveolar sarcoma. Tissue culture. " Chief " cells in unextended clumps. ×1000. Phase.
FIGS. 12, 13.—Alveolar sarcoma. Tissue culture. " Chief " cells extended on glass. ×1000. Note opaque cytoplasm and curved culture of cytoplasm.
FIG. 14.—Alveolar sarcoma. Tissue culture. " Chief " cells, ×1000. Wigglesworth's method. Lipoid granules marked, but the bulk of the cytoplasmic matter is not of lipoid nature.

Udekwu and Pulvertaft.

5

6

7

8

9

10

11

12

13 14

Udekwu and Pulvertaft.

trypsin is not satisfactory and collagenase is used ; but in this case the material rapidly became mucinous and when well agitated and filtered through one layer of gauze a very satisfactory cell suspension was achieved.

The medium consisted of 20 % adult human serum, 2 % bovine embryo extract and 78 % filtered human asctic fluid from a case of nephrosis. Nystatin and neomycin were added.

While ascitic fluid, undiluted, from cases of cardiac or hepatic ascites, is not satisfactory, the peculiar fluid found in juvenile nephrosis in Nigeria is an excellent balanced and buffered salt solution of human origin. Since many litres are removed on each occasion there is ample medium for each experiment. It is quite colourless, contains no fibrinogen and in fact yields no coagulation on boiling.

Control cultures from a haemangioma of the nasal cavity were prepared at the same time. The fibrinoblasts and mast cells grown are shown by phase contrast (Fig. 5) and stained by Wigglesworth's (1959) method (Fig. 5) for comparison.

The cultures were very successful and all the dispersed cells adhered to the glass cover slip in the ring chambers. They were examined and photographed at 37° C.

The culture was a mixed one, a proportion of the cells being normal fibroblasts, which alone multiplied and eventually swamped the cultures.

The most unusual cells were of fibroblastic form, but were packed with large spherical granules, very dark by phase contrast and brown by direct light (Fig. 7, 8, 9). These cells were only seen in early cultures and apparently died. The chemical composition of the spherical granules is therefore not known. Fig. 10 shown a Wigglesworth stain of an early culture. At the edge of the cytoplasm clear spherical bodies are seen which are not of lipoid origin.

The " chief cells " remained in rounded three dimensional clumps for the most part during a 14-day culture period and did not for the most part flatten on the glass (Fig. 11). Their cytoplasm was very dense and the structure difficult to observe. A minority did spread, however (Fig. 12) and showed a very characteristic appearance. The nucleus was spherical, with a pronounced nucleolus. The cytoplasm always showed a curve instead of the normal sharp angle of a fibroblast. It was densely packed with granules of lipoid nature (Fig. 14), but in addition showed phase contrast opacity not resolvable into granules. Chromaffin staining gave a negative result.

In order to see whether the cells would react to a high concentration of $CO_2$ the culture medium was made acid by bubbling this gas through it ; the cells did not change in any way. Cells were also grown in a medium from which all dissolved gases were removed by exhaustion with a mercury pump ; the medium was then saturated with 5 % $CO_2$ in pure nitrogen. Again the cells showed no change over a 24 hour period. It is not known whether tissue cultures of a chemodectoma such as a carotid body tumour would react to this type of treatment which would perhaps be expected ; the cells from this tumour however did not.

After about 14 days the cultures were overgrown by fibroblasts and the experiment was terminated. The " chief cells " had thoughout shown no mitosis.

### DISCUSSION

The histology of a true chemodectoma and its appearances in tissue culture have been excellently displayed by Costero (1963). In a profusely illustrated paper the author states that four types of cells can be recognized : (1) " chief cells ", (2) nerve fibrils, (3) cells containing silver reducing substances and (4) synaptic complexes. Mast cells were said to be numerous ; in our case there were none. The illustrations of material stained by Hortega's method show clearly pyriform unipolar cells in alveolar aggregates ; these were absent in our case.

Costero's cultures showed oligodendroglia, with delicate pulsating filopodia, and amoeboid granular cells, both absent in our case.

While these tissue culture studies show that the alveolar soft-part sarcoma and the chemodectoma are entirely different, facilities were not available for histochemical tests on the " chief cells " in culture. The cytoplasmic granularity is not due to lipoid material; in fact the phase positive material in the cytoplasm seems to be of non-particulate nature. Fisher (1956) has analysed the available literature and came to the conclusion that the granular material was a cerebroside. Since the " chief cells " of this tumour can readily be dispersed by trypsin and maintained in short term culture confirmation of this in suitably equipped laboratories should be simple.

The belief that certain soft tissue tumours are in fact non-chromaffin paraganglia was first explicitly stated by Smetana and Scott (1951). From their descriptions it seems clear that their material certainly included cases similar to the present case. Tissue culture studies were not made and Hortega's stain was not used, although nerve fibres were noted. It was specifically stated that direct relationship between nerve fibres and tumour cells was not investigated.

SUMMARY

A case of alveolar soft tissue sarcoma in a young Nigerian male is reported. Tissue cultures following trypsin dispersal were readily made. These contrasted strongly with tissue cultures of chemodectomas from the carotid body reported by Costero (1963). It is concluded that the alveolar soft-part sarcoma is not a chemodectoma.

Our thanks are due to Professor G. M. Edington for much help, particularly in drawing our attention to the recent work of Costero (1963), and to Professor D. F. Cappell for criticism. The photographic prints were made by the Medical Illustration Department, University of Ibadan. The technical work was done by Mrs. Elizabeth Pulvertaft.

The expenses of this investigation were covered by a full time grant from the British Empire Cancer Campaign for Research to one of us (R.J.V.P.).

### REFERENCES

Christopherson, W. M., Foote, F. W. and Stewart, F. W.—(1952) *Cancer, N.Y.*, **5**, 100.

Costero, I.—(1963) *Lab. Invest.*, **12**, 270.

Fisher, E. R.—(1956) *Am. J. Path.*, **32**, 721.

Pack, G. T. and Ariel, I. M.—(1958) 'Tumours of the Soft Somatic Tissues'. New York (Hoeber-Harper & Co.).

Smetana, H. F. and Scott, W. F.—(1951) *Milit. Surg.*, **109**, 330.

Wigglesworth, V. B.—(1959) *Q. Jl microsc. Sci.*, **100**, 315.


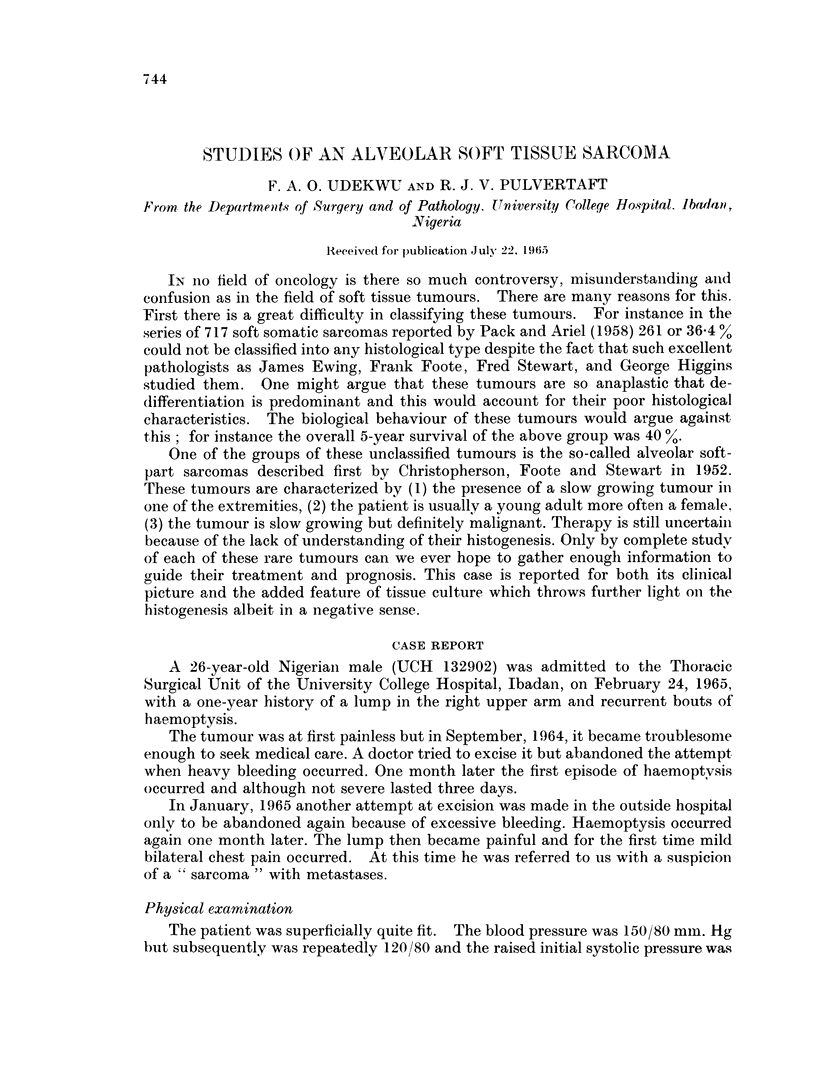

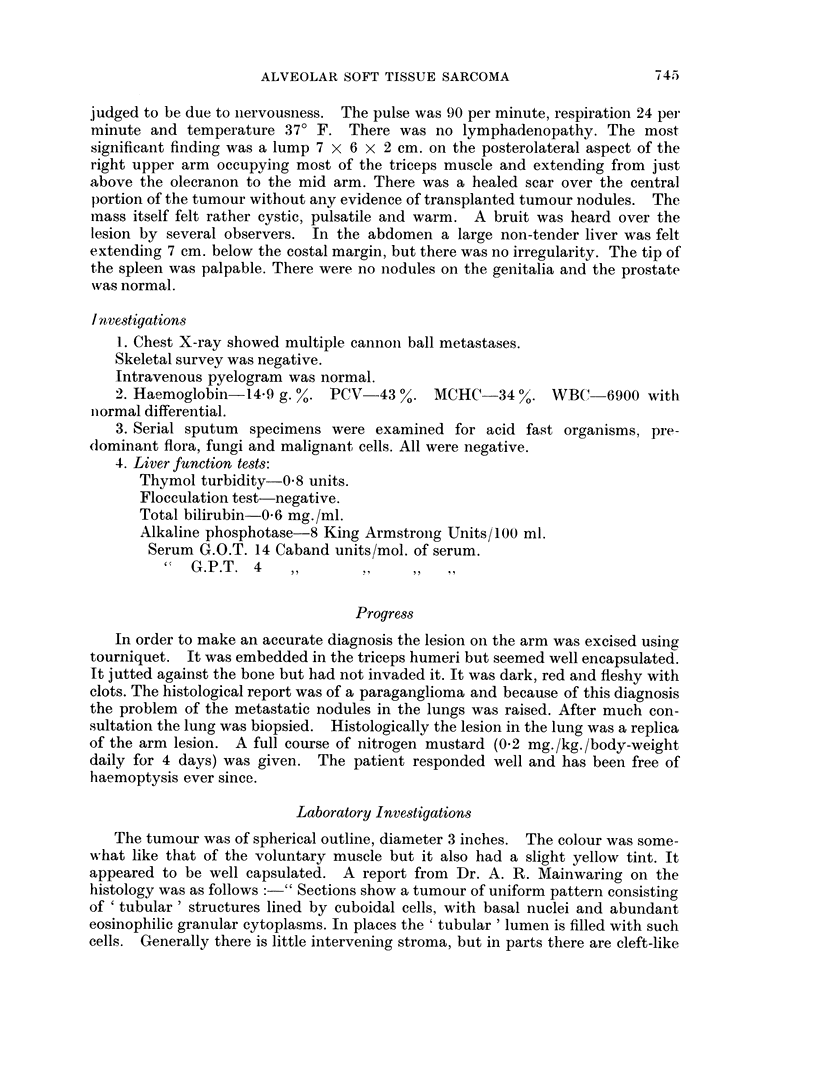

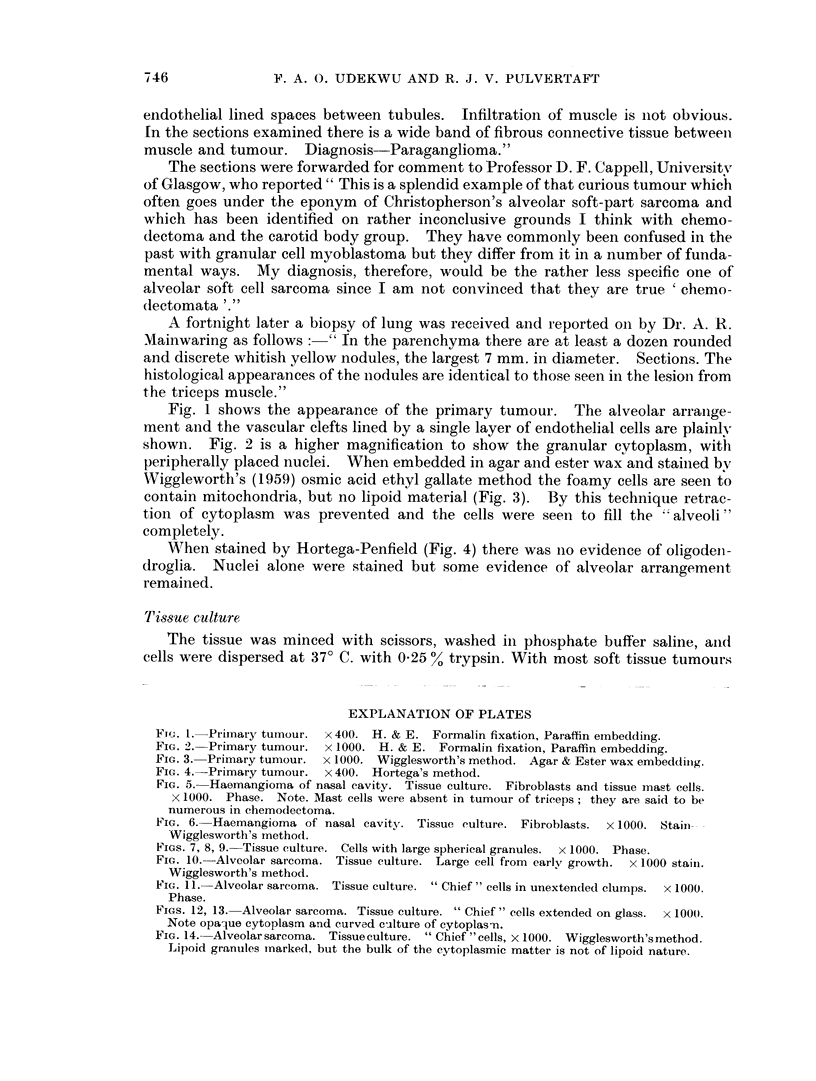

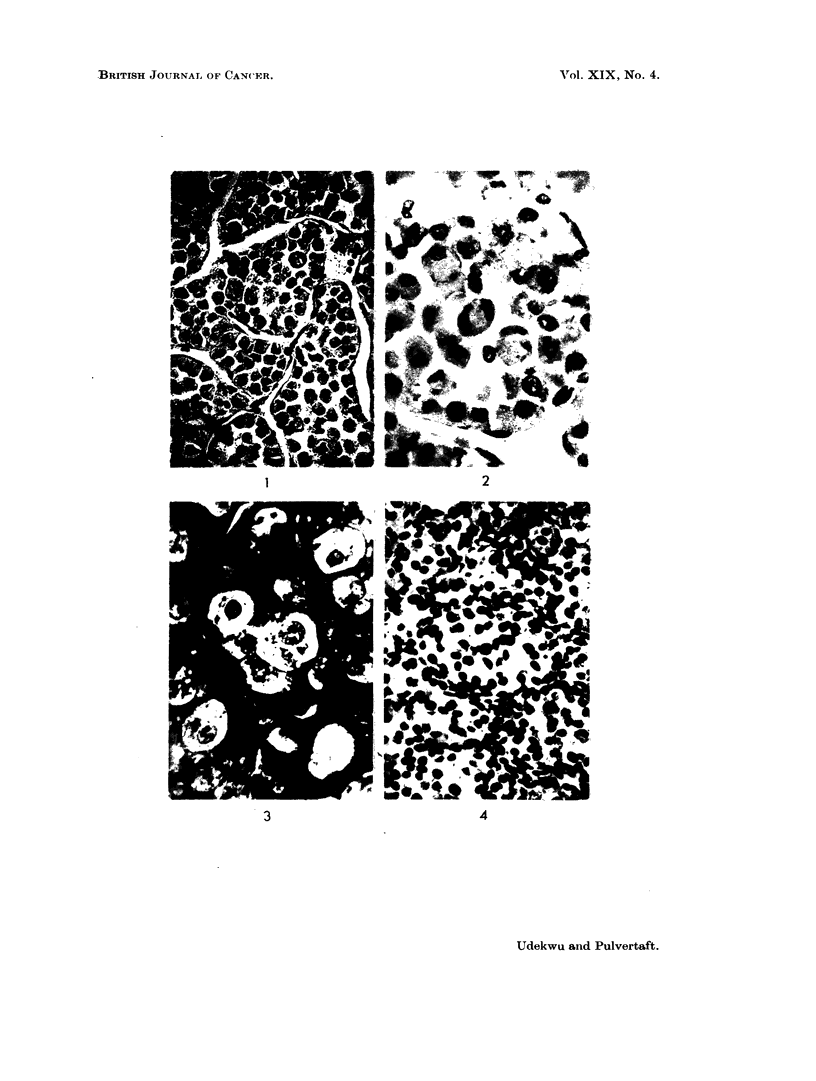

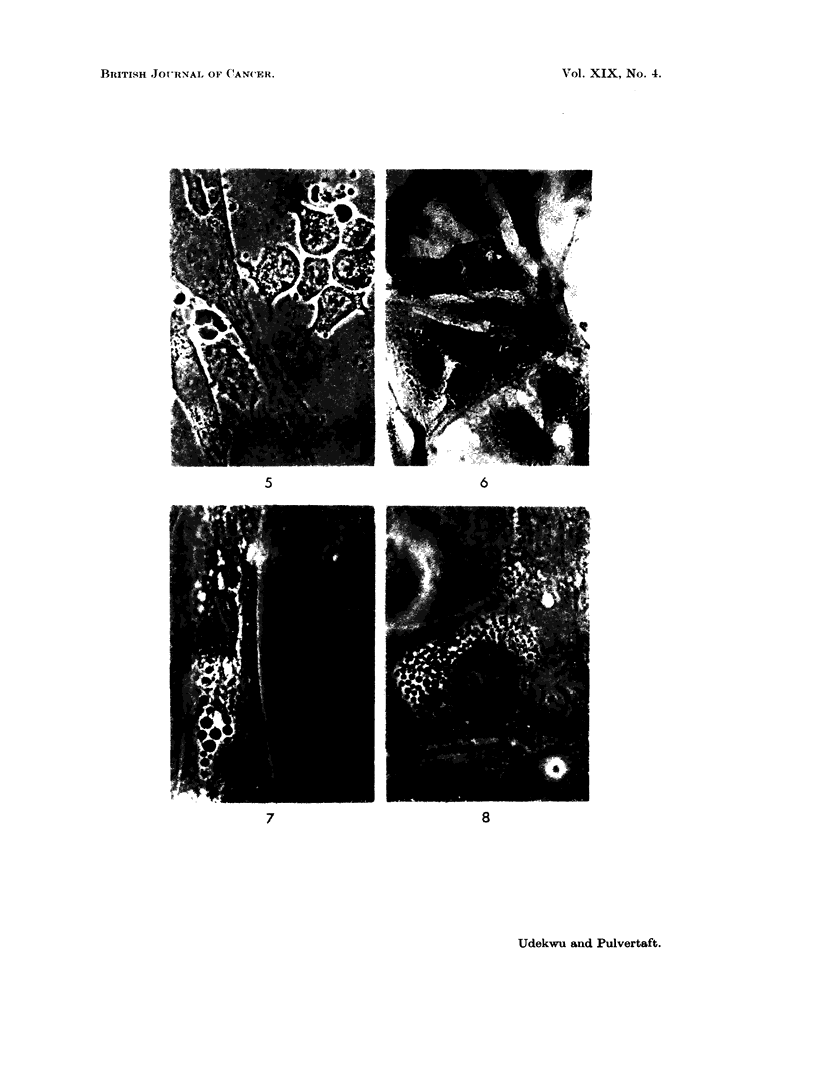

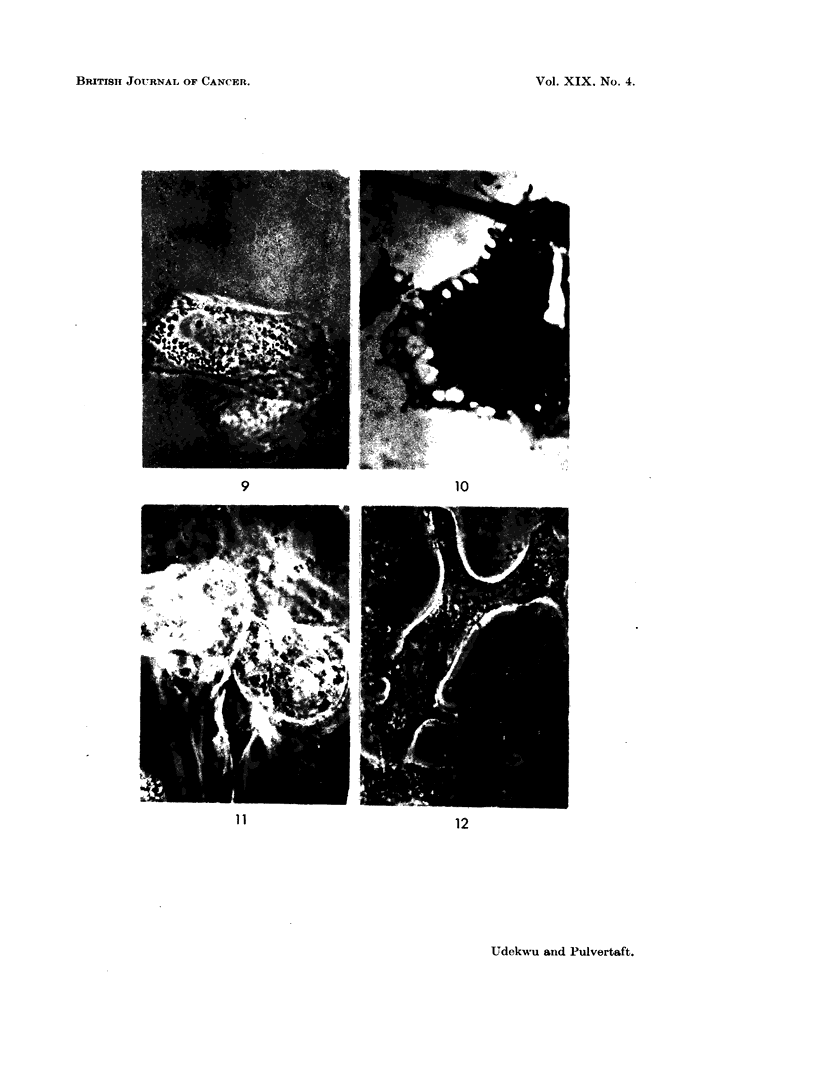

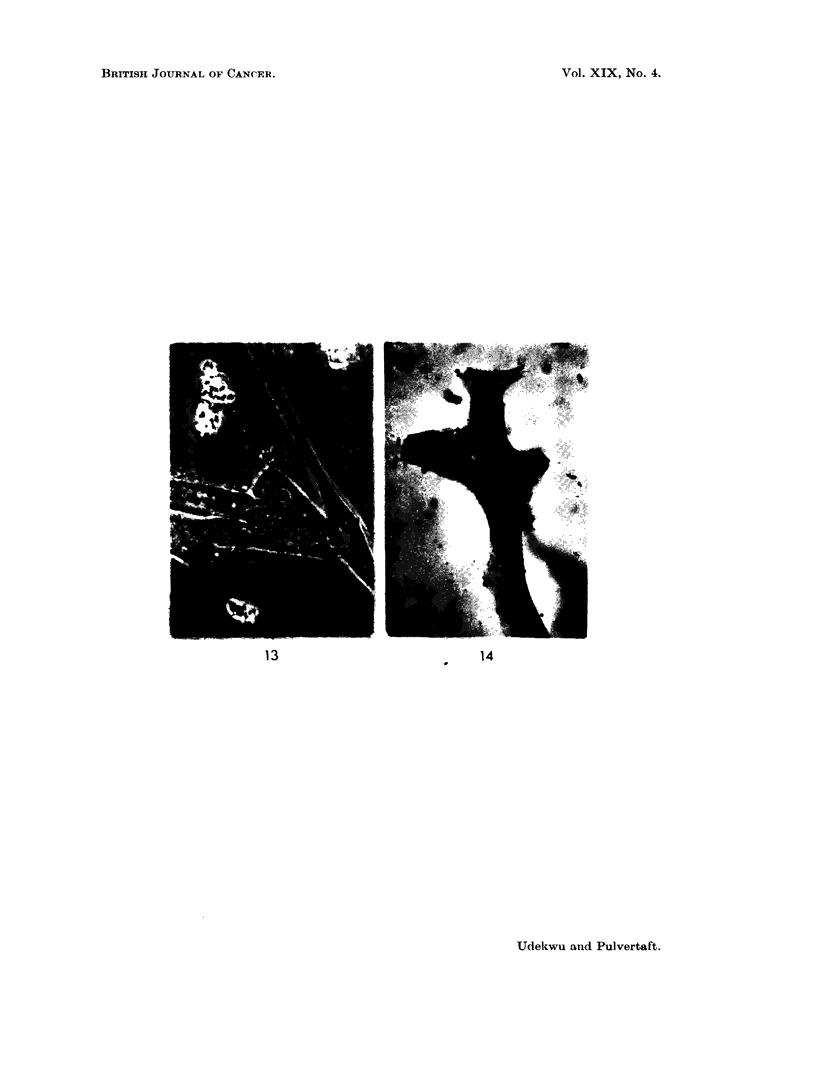

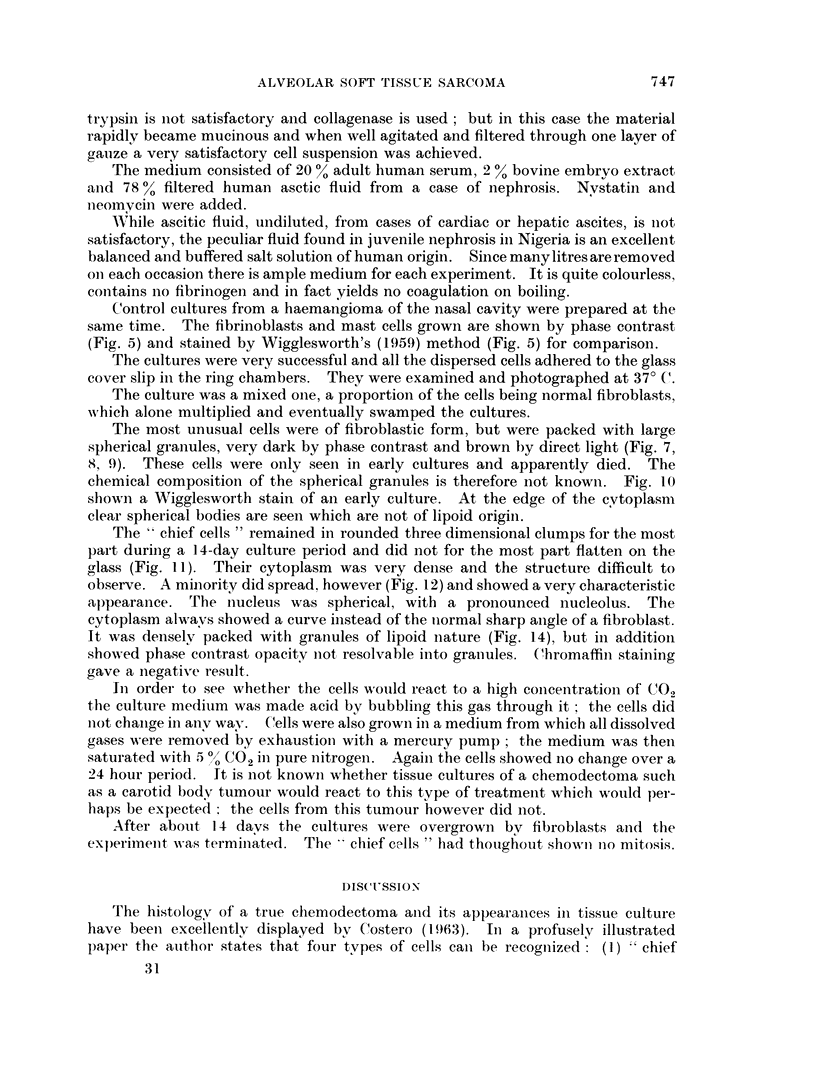

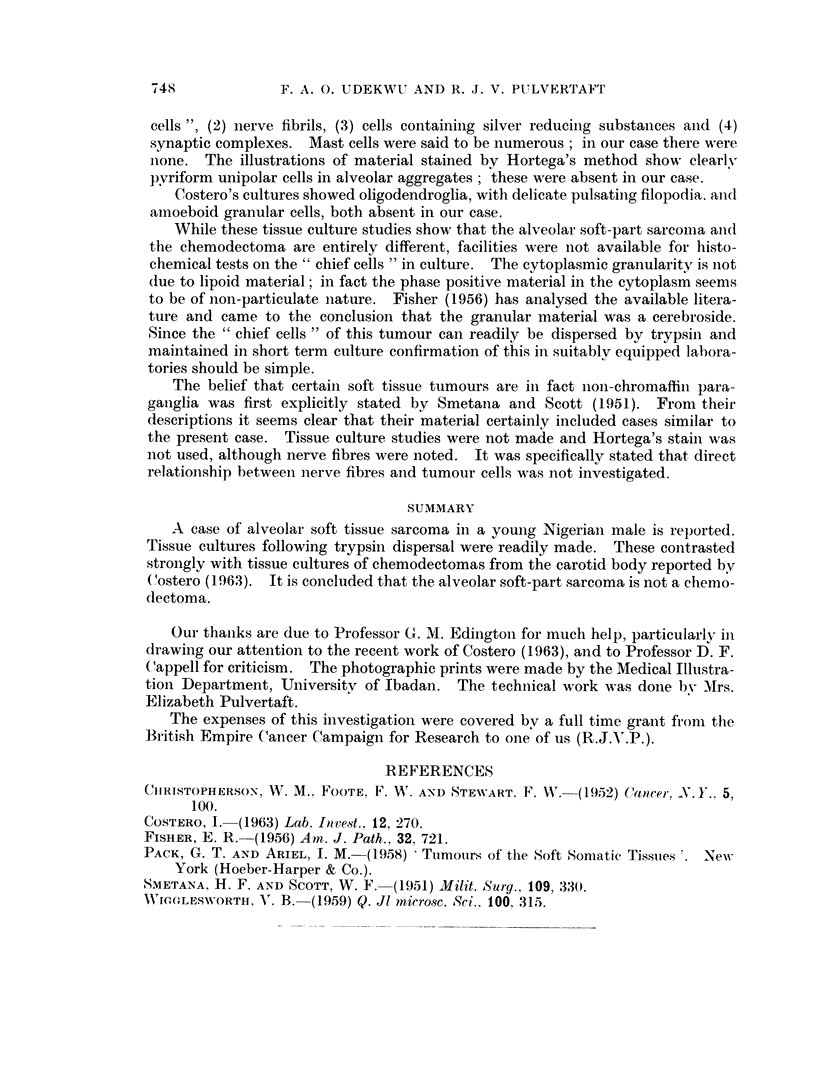


## References

[OCR_00388] COSTERO I. (1963). Recent advances in the knowledge concerning chemodectomas.. Lab Invest.

[OCR_00390] FISHER E. R. (1956). Histochemical observations on an alveolar soft-part sarcoma with reference to histogenesis.. Am J Pathol.

[OCR_00396] SMETANA H. F., SCOTT W. F. (1951). Malignant tumors of nonchromaffin paraganglia.. Mil Surg.

